# TGF-β1-induced epithelial–mesenchymal transition in lung cancer cells involves upregulation of miR-9 and downregulation of its target, E-cadherin

**DOI:** 10.1186/s11658-017-0053-1

**Published:** 2017-11-02

**Authors:** Hui Wang, Qian Wu, Ying Zhang, Hua-Nan Zhang, Yong-Bin Wang, Wei Wang

**Affiliations:** Department of Respiratory Medicine, The Second Hospital of Shandong University, 247 Beiyuan Street, Jinan, Shandong 250033 China

**Keywords:** MiR-9, E-cadherin, Invasion, NSCLC

## Abstract

**Background:**

TGF-β1 plays an important role in the epithelial–mesenchymal transition (EMT) of epithelial cancers, including non-small cell lung cancer (NSCLC). While the full underlying mechanism remains unclear, miR-9 is known to play a critical role in the regulation of NSCLC cell invasion. We tested whether miR-9 targets E-cadherin and thus affects TGF-β1-induced EMT in NSCLC cells by assessing the expression levels of miR-9 and E-cadherin for NSCLC patients and then verifying the targeting of E-cadherin by miR-9 using the dual luciferase reporter system.

**Results:**

MiR-9 was significantly upregulated in NSCLC tissues compared with its level in adjacent normal tissues. The expression of E-cadherin in NSCLC tissues was significantly decreased. In addition, we found that TGF-β1 significantly upregulated the expression of miR-9 and downregulated the expression of E-cadherin. E-cadherin was confirmed as a direct target gene of miR-9. Using an miR-9 inhibitor reversed the TGF-β1-mediated inhibition of E-cadherin expression and upregulation of the mesenchymal marker α-SMA. TGF-β1 significantly induced cell invasion, and this effect was significantly inhibited by miR-9 inhibitors.

**Conclusions:**

TGF-β1 induced EMT in NSCLC cells by upregulating miR-9 and downregulating miR-9’s target, E-cadherin.

## Background

Lung cancer, which ranks first in global cancer-related mortality rates, can be divided into two main types: non-small cell lung cancer (NSCLC), which accounts for almost 80% of cases [1–4], and small cell lung cancer, which accounts for about 20% [5, 6]. Modern treatments mainly rely on radiotherapy and chemotherapy [7].

Recent studies have shown that microRNAs (miRNAs) are of great value in the early diagnosis and treatment of NSCLC [8], making it particularly important to identify effective miRNAs and elucidate their molecular mechanisms.

MiRNAs are highly conserved non-coding RNA with approximately 18~24 NTs. They are involved in gene regulation, acting by binding to 3′ untranslated regions (UTRs) of target mRNA [9–11]. The important biological processes involving miRNAs include the development, differentiation, proliferation and apoptosis of cells [9, 12, 13]. One important example, miR-9 has been widely found in many different species. It is involved in regulating the development of organisms and cell self-renewal, differentiation, and many other physiological activities.

Abnormal miRNA expression is usually associated with inhibition or progression of cancer. Studies have shown that miRNAs serve as tumor suppressor or oncogenes [14, 15]. The miRNA microarray analysis of lung cancer and adjacent normal tissues have shown that miR-9 is upregulated in lung cancer tissues in early developmental stages [16] and miRNA expression array assays have confirmed its overexpression [17], which is closely associated with adverse clinical features and unfavorable survival. Thus, miR-9 is a biomarker of poor prognosis in NSCLC patients [18].

It was recently demonstrated that SRY-Box7 is a direct target of miR-9 [18]. MiR-9 expression negatively correlates with SRY-Box7 expression in human NSCLC [18]. Moreover, miR-9 is upregulated by TGF-β1 and contributes to TGF-β1-induced NSCLC cell invasion through directly targeting of SRY-Box7 [18]. However, the role of miR-9 in the regulation of NSCLC cell invasion and the underlying molecular mechanisms remain unclear.

TGF-β1 plays an important role in the induction of epithelial–mesenchymal transition (EMT). Recent studies have shown that EMT of epithelial cancer cells including A549 and hepG2 are regulated by TGF-β1 autocrine, contributing to pulmonary fibrosis or hepatocellular carcinoma metastasis [19, 20]. TGF-β1 induced a phenotype transition in cells. After TGF-β1 treatment, cells become more migratory and less adhesive. Moreover, TGF-β1 downregulates E-cadherin (a marker for the epithelial phenotype) and upregulates α-SMA (a marker for the mesenchymal phenotype).

We predicted that E-cadherin might be the target gene of miR-9 and hypothesized that the interaction of miR-9 and E-cadherin plays an important role in the EMT of NSCLC cells. To investigate their role in the EMT of NSCLC cells, we studied the expression levels of miR-9 and E-cadherin in NSCLC patients, and then verified the targeting of E-cadherin by miR-9. Our results show that miR-9 is indeed involved in TGF-β1-induced EMT of NSCLC and the mechanism involves direct targeting of E-cadherin.

## Methods

### NSCLC patients and tissue samples

The study was approved by the Ethics Committee of the Second Hospital of Shandong University and carried out according to the World Medical Association Declaration of Helsinki. All patients were enrolled after giving written informed consent. Cancer tissue samples and matched adjacent non-cancerous tissue samples were collected from 20 NSCLC patients (14 male and 6 female, with a median age of 49 years).

### Cell culture and transfection

The cell lines A549, NCI-H1299 and HCC827 were purchased from the Cell Bank of the Chinese Academy of Sciences in Shanghai, and HEK293 and normal human bronchial epithelial (HBE) were purchased from the American Tissue Culture Collection. All cells were cultured in DMEM (Invitrogen) containing 10% fetal bovine serum (FBS; Invitrogen) and maintained at 37 °C in a humidified atmosphere containing 5% CO_2_. Cells were treated with 5 ng/ml TGF-β1 (PeproTech) for 24 h.

The miR-9 mimics, inhibitor and negative control (NC) were purchased from Biomics Biotech. miR-9 mimics are small, chemically modified double-stranded RNAs that mimic endogenous miRNA-9. miR-9 inhibitors are small, chemically modified double-stranded RNAs designed to specifically bind to and inhibit endogenous miRNA-9.

A549 cells were seeded in a 6-well plate at 2.5 × 10^5^ cells/well) for 24 h before transfection, and then were transfected with miR-9 mimics (50 nM), inhibitor (50 nM) or NC (50 nM) using Lipofectamine 2000 (Invitrogen) in accordance with the manufacturer’s instructions. After transfection, the cells were grown in medium without antibiotics for 48 h and the expressions of miR-9 and E-cadherin in transfected cells were detected. Cells were then used for the following experiments.

### Quantitative RT-PCR

Total RNA was extracted from the tissues and cells using RNeasy and miRNeasy Kits (Qiagen). One μg of total RNA was reverse-transcribed to cDNA using the Superscript II Reverse Transcriptase Kit (Invitrogen), and reverse transcription reaction were performed according to the manufacturer’s instructions. PCR was performed on an Applied Biosystems 7500 Sequence Detection System using a SYBR Premix Ex Taq GC kit (Takara). The primers were: miR-9: 5′-TCTTTGGTTATCTAGCTGTATGA-3′ (sense), 5′-TGGTGTCGTGGAGTCG-3′ (antisense); U6: 5′-CTCGCTTCGGCAGCACA -3′ (sense), 5′-AACGCTTCACGAATTTGCGT-3′ (antisense); E-cadherin: 5′-AAAGGCCCATTTCCTAAAAACCT-3′ (sense), 5′-TGCGTTCTCTATCCAGAGGCT-3′ (antisense); GAPDH: 5′-CGACCACTTTGTCAAGCTCA-3′(sense), 5′-AGGGGAGATTCAGTGTGGTG-3′ (antisense). The gene expression was normalized to the level of GAPDH using the relative ^ΔΔ^C_T_ method.

### Cell invasion assay

Cell invasion was performed using Transwell inserts with 8 μm membrane (Corning) precoated with Matrigel (BD) seeded with 1 × 10^5^ cells/well in the upper chamber. Medium containing 10% FBS was added to the lower chamber. Following incubation for 24 h, the non-invaded cells were removed with a swab cotton, and the invaded cells on the surface of the membrane were fixed in ethanol, stained with hematoxylin, imaged, and counted under a microscope.

### Luciferase reporter assay

The wild-type and mutant E-cadherin 3’UTR sequences were amplified using PCR and ligated into the PMIRREPORT luciferase vector (Ambio) to yield pMIR-E-cadherin 3’UTR (E-cad 3’UTR). HEK293 cells were seeded into 6-well plates and co-transfected with wild-type or mutant E-cad 3’UTR and miR-9 mimics or NC using Lipofectamine 2000. After 24 h, the cells were harvested and lysed, and the luciferase reporter assay was carried out according to the manufacturer’s instructions (Promega).

### Western blotting

Total proteins were extracted from NSCLC tissues and cell lines using ice-cold radioimmune precipitation (RIPA) buffer. Protein concentration was quantified using the Bio-Rad kit (Bio-Rad). 50–100 μg of proteins were separated using 10% SDS–PAGE gel and transferred to nitrocellulose membranes (Millipore). Membranes were blocked with 5% non-fat milk in TBS-Tween and incubated with primary antibody against E-cadherin (1:500; Santa Cruz Biotechnology) and GAPDH (1:1000; Santa Cruz Biotechnology) overnight at 4 °C, followed by incubation with HPR-conjugated secondary antibody (Santa Cruz Biotechnology) for 60 min at room temperature. Polyclonal anti-GAPDH was used as an internal control. Then, the proteins were visualized using the ECL detection system (Amersham Pharmacia Biotech).

### Statistical analysis

All statistical analyses were performed using SPSS version 19.0 (SPSS Inc.) and Student’s *t*-test, except for the coefficients of correlation, which were determined using Pearson’s correlation methods [20]. The data are presented as the means ± SD. *p* < 0.05 is considered statistically significant.

## Results

### MiR-9 was upregulated and E-cadherin was downregulated in NSCLC tissues

**Fig. 1:**
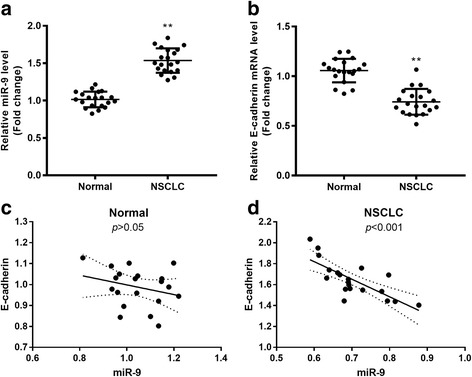
Expression of miR-9 and E-cadherin in human NSCLC. In human NSCLC tissue and adjacent normal tissue, the relative expressions of miR-9 (**a**) and E-cadherin mRNA (**b**) were assessed with qRT-PCR. **p < 0.01 vs. normal control. The correlations between miR-9 and E-cadherin in adjacent normal tissues (**c**) and NSCLC cancer tissues (**d**) were determined using Pearson analyses

Paired samples of NSCLC cancer tissue and adjacent non-cancerous tissue were collected (*n* = 20), and the expressions of miR-9 and E-cadherin mRNA were assessed via qRT-PCR (Fig. 1). The results reveled that miR-9 was significantly upregulated in the NSCLC tissues compared with the level in normal tissues (*p* < 0.01; Fig. 1a). The E-cadherin mRNA in NSCLC tissues was significantly decreased (*p* < 0.01; Fig. 1b). These data suggested that miR-9 is significantly downregulated and E-cadherin is significantly upregulated in patients with NSCLC.

A correlation analysis between miR-9 and E-Cadherin in primary NSCLC tissues and adjacent non-cancerous tissues was performed (Fig. 1c and d). There was a negative association between miR-9 and E-Cadherin in NSCLC tissue. Thus, miR-9 was negatively associated with E-Cadherin in NSCLC.

### Expression of miR-9 and E-cadherin in NSCLC cell lines

**Fig. 2:**
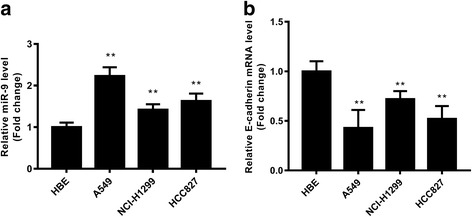
Expression of miR-9 and E-cadherin in NSCLC cell lines. Relative expression of miR-9 (**a**) and E-cadherin mRNA (**b**) were assessed with qRT-PCR. *p < 0.01 vs. HBE cells

To test the roles of miR-9 and E-cadherin in NSCLC, we used the A549, HCI-H1299 and HCC827 cell lines with the normal HBE line as a control. The results showed that miR-9 expression was significantly upregulated in all three NSCLC cell lines, while E-cadherin mRNA was significantly downregulated in all the three (Fig. 2). This suggests that miR-9 expression is inversely correlated with E-cadherin expression in NSCLC cell lines. A549, which has high endogenous miR-9 expression and low endogenous E-cadherin expression, was selected for subsequent experiments.

### TGF-β1 upregulated the expression of miR-9 and downregulated the expression of E-cadherin in A549 cells

**Fig. 3:**
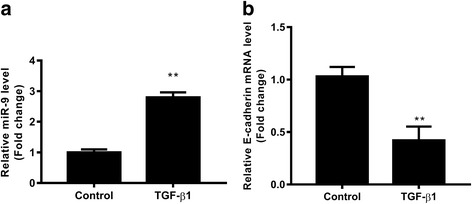
TGF-β1 promotes the expression of miR-9 and inhibits the expression of E-cadherin mRNA. After treatment with TGF-β1 (5 ng/ml) for 24 h, the expressions of miR-9 (**a**) and E-cadherin mRNA (**b**) in A549 cells were detected using qRT-PCR. Control cells without TGF-β1 treatment. *p < 0.01 vs. control

To investigate the effect of miR-9 on TGF-β1-induced NSCLC cell invasion, the levels of miR-9 and E-cadherin in A549 cells were determined. TGF-β1 significantly upregulated the expression of miR-9 and downregulated the expression of E-cadherin mRNA (Fig. 3), indicating the miR-9 and E-cadherin play an important role in TGF-β1-induced NSCLC cell invasion.

### E-cadherin is a direct target of miR-9

**Fig. 4:**
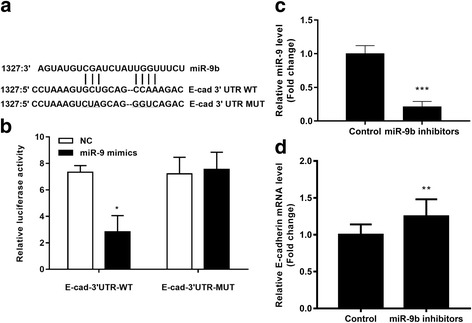
miR-9 directly targets E-cadherin. **a** – Bioinformatics prediction of the binding site of miR-9 with E-cadherin 3’UTR. **b** – The relative luciferase activity of E-cadherin wild-type (WT) or mutant (MUT) 3’UTR in HEK293 cells following transfection with miR-9 mimics. **c** – Relative expression of miR-9 in A549 cells transfected with miR-9 inhibitors. **d** – Relative expression of E-cadherin mRNA in A549 cells transfected with miR-9 inhibitors. *p < 0.01 vs. WT or NC

We investigated the interaction site of miR-9 and E-cadherin 3’UTR. The predicted binding sequences of wild-type and mutant E-cadherin 3’UTR are shown in Fig. 4a. They were transfected into HEK293 cells together with miR-9 mimics or NC. The results of luciferase reporter assays showed that the transcriptional activity of wild-type E-cadherin 3’UTR was significantly decreased by miR-9 mimics. The transcriptional activity of mutant E-cadherin 3’URT was not affected by miR-9 mimics (Fig. 4b).

The roles of miR-9 in E-cadherin were confirmed by transfecting the miR-9 inhibitors into A549 cells. The results of qRT-PCR analysis demonstrated that miR-9 was significantly downregulated in miR-9 inhibitor-transfected cells (Fig. 4d), and that E-cadherin was also significantly overexpressed in cells transfected with the miR-9 inhibitor (Fig. 4d).

### MiR-9 inhibitor suppressed TGF-β1-induced EMT in NSCLC cells

**Fig. 5:**
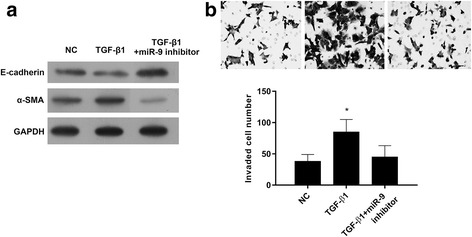
miR-9 inhibitors inhibit TGF-β1-induced EMT. After transfection with miR-9 inhibitors for 24 h, the cells were treated with TGF-β1 (5 ng/ml) for 24 h. **a** – The expression levels of α-SMA and E-cadherin were detected using western blot. **b** – Cell invasion was detected using a Transwell assay. *p < 0.01 vs. control

MiR-9 inhibitor reversed the TGF-β1-inhibited expression of the epithelial marker E-cadherin and suppressed the TGF-β1-induced expression of the mesenchymal marker α-SMA (Fig. 5a). Cell invasion was significantly induced by TGF-β1, and this effect was significantly inhibited by miR-9 inhibitors (Fig. 5b). These results suggest that miR-9 inhibitor inhibits TGF-β1-induced EMT in NSCLC cells.

## Discussion

We showed that miR-9 is involved in TGF-β1-induced EMT of lung cancer cell due to its direct targeting of E-cadherin. Originally identified in the nervous system, miR-9 regulates neurodevelopment through its role in nerve or non-neural cell lines by targeting the 3 ‘UTR of various genes. It was recently demonstrated that miR-9 is irregularly expressed in cancers including head and neck squamous cell carcinoma (HNSCC) [21], bladder cancer [22], papillary thyroid cancer [23], and NSCLC [24]. miR-9 was expressed at high levels in recurrent HNSCC, where it targeted SASH1 and KRT13 [21]. However, the role of miR-9 in the NSCLC was not clear.

Abnormalities in miR-9 levels have been reported for various types of cancer, suggesting that miR-9 is involved in tumor formation and progression. It plays a different role in the proliferation of various types of tumor cells through its regulation of a variety of target gene mRNAs. For example, miR-9 is overexpressed in human Hodgkin’s lymphoma cells and primary brain tumor cells [25–27]. The decrease in miR-9 expression levels due to methylation is associated with metastatic tumor cells in lymph nodes [28, 29], and is associated with metastatic recurrence of patients with clear renal cell carcinoma [30]. By contrast, miR-9 expression levels are significantly higher in metastatic breast cancer than in non-metastatic, and miR-9 appears to promote tumor metastasis [31].

MiR-9 induces EMT in human epithelial cells and breast cancer cell lines by regulating the adhesion protein E-cadherin [31]. Cells overexpressing miR-9 lose contact with each other and their movement and migration are enhanced in vitro. MiR-9 also induces the transcription of vascular endothelial growth factor (VEGF) by downregulation of E-cadherin, leading to the release of β-catenin from the junction into the nucleus. However, other miR-9 related target genes may also increase VEGF transcription and miR-9-induced migration, since E-cadherin knockdown is not able to mimic the in vitro miR-9 overexpression phenotype of breast cancer cells [31].

When breast cancer cells overexpressing miR-9 were implanted into mice, the angiogenesis in the tumor was markedly enhanced and the tumor grew faster than the cells with low miR-9 expression. These findings suggest that miR-9 promotes metastasis of epithelial tumors. In fact, overexpression of miR-9 in non-metastatic breast tumors favors the formation of small metastases in the lungs, while knockout of miR-9 inhibits the transfer of highly metastatic cells [31].

Previous studies showed that miR-9 was upregulated in lung cancer tissues compared with the adjacent normal lung tissues [18, 32–35]. Overexpression of miR-9 is a poor prognosis biomarker in NSCLC patients [18]. Our results also showed that miR-9 was upregulated in NSCLC tissues.

It was recently demonstrated that SRY-Box7 was a direct target of miR-9 [18]. MiR-9 expression was negatively correlated with SRY-Box7 expression in human NSCLC, and miR-9 was upregulated by TGF-β1 and contributed to TGF-β1-induced NSCLC cell invasion by directly targeting SRY-Box7 [18].

Here, we found that TGF-β1 plays an important role in EMT. TGF-β1 induces a phenotype transition, becoming more migratory and less adhesive through downregulation of the E-cadherin marker for the epithelial phenotype and upregulation of the α-SMA marker for the mesenchymal phenotype. As shown in Fig. 5, TGF-β1 suppressed the expression of E-cadherin, while miR-9 inhibitor increased the expression of E-cadherin after TGF-β1 treatment, suggesting miR-9 is involved in the role of TGF-β1 in NSCLC. Consistently with this, miR-9 inhibitor greatly reduced TGF-β1-induced NSCLC cell invasion. Thus, we have shown that TGF-β1 induces NSCLC cell invasion through upregulation of miR-9 and downregulation of miR-9’s target, E-cadherin.

## Conclusion

MiR-9 is involved in TGF-β1-induced EMT of NSCLC through its direct targeting of E-cadherin. Our findings reveal that miR-9 suppresses E-cadherin expression, but further investigation of the mechanism is required. miR-9 may be a diagnostic marker and a new therapeutic target in NSCLC. Further studies are required to analyze the association between miR-9 and NSCLC.

## Abbreviations

EMT: Epithelial-mesenchymal transition
HBE: Human bronchial epithelial
MiRNAs: microRNA
NSCLC: Non-small cell lung cancer
UTRs: Untranslated regions
VEGF: Vascular endothelial growth factor

## References

[1] Pan X, Zhang X, Sun H, Zhang J, Yan M, Zhang H (2013). Autophagy inhibition promotes 5-fluorouraci-induced apoptosis by stimulating ROS formation in human non-small cell lung cancer A549 cells. PLoS One.

[2] Chiu LY, ME H, Yang TY, Hsin IL, Ko JL, Tsai KJ, Sheu GT (2015). Immunomodulatory protein from Ganoderma microsporum induces pro-death autophagy through Akt-mTOR-p70S6K pathway inhibition in multidrug resistant lung cancer cells. PLoS One.

[3] Hsin IL, Sheu GT, Jan MS, Sun HL, TC W, Chiu LY, Lue KH, Ko JL (2012). Inhibition of lysosome degradation on autophagosome formation and responses to GMI, an immunomodulatory protein from Ganoderma microsporum. Br J Pharmacol.

[4] Hsin IL, CC O, TC W, Jan MS, MF W, Chiu LY, Lue KH, Ko JLGMI (2011). An immunomodulatory protein from Ganoderma microsporum, induces autophagy in non-small cell lung cancer cells. Autophagy.

[5] Zhu L, Pickle LW, Ghosh K, Naishadham D, Portier K, Chen HS, Kim HJ, Zou Z, Cucinelli J, Kohler B (2012). Predicting US- and state-level cancer counts for the current calendar year: part II: evaluation of spatiotemporal projection methods for incidence. Cancer.

[6] Chen HS, Portier K, Ghosh K, Naishadham D, Kim HJ, Zhu L, Pickle LW, Krapcho M, Scoppa S, Jemal A (2012). Predicting US- and state-level cancer counts for the current calendar year: part I: evaluation of temporal projection methods for mortality. Cancer.

[7] Seifi-Najmi M, Hajivalili M, Safaralizadeh R, Sadreddini S, Esmaeili S, Razavi R, Ahmadi M, Mikaeili H, Baradaran B, Shams-Asenjan K (2016). SiRNA/DOX lodeded chitosan based nanoparticles: development, characterization and in vitro evaluation on A549 lung cancer cell line. Cell Mol Biol (Noisy-le-grand).

[8] Li J, Yi W, Jiang P, Sun R, Li T (2016). Effects of ambroxol hydrochloride on concentrations of paclitaxel and carboplatin in lung cancer patients at different administration times. Cell Mol Biol (Noisy-le-grand).

[9] Bartel DP (2004). MicroRNAs: genomics, biogenesis, mechanism, and function. Cell.

[10] Rajewsky N (2006). microRNA target predictions in animals. Nat Genet.

[11] Valencia-Sanchez MA, Liu J, Hannon GJ, Parker R (2006). Control of translation and mRNA degradation by miRNAs and siRNAs. Genes Dev.

[12] Rosa A, Brivanlou AH (2009). MicroRNAs in early vertebrate development. Cell Cycle.

[13] Harfe BD (2005). MicroRNAs in vertebrate development. Curr Opin Genet Dev.

[14] Fattore L, Costantini S, Malpicci D, Ruggiero CF, Ascierto PA, Croce CM, Mancini R, Ciliberto G. MicroRNAs in melanoma development and resistance to target therapy. Oncotarget. 2017;8(13):22262–78. 10.18632/oncotarget.14763 PMC5400662 28118616

[15] Lin X, Khalid S, Qureshi MZ, Attar R, Yaylim I, Ucak I, Yaqub A, Fayyaz S, Farooqi AA, Ismail MVEGF (2016). Mediated signaling in oral cancer. Cell Mol Biol (Noisy-le-grand).

[16] Volinia S, Calin GA, Liu CG, Ambs S, Cimmino A, Petrocca F, Visone R, Iorio M, Roldo C, Ferracin M (2006). A microRNA expression signature of human solid tumors defines cancer gene targets. Proc Natl Acad Sci U S A.

[17] Vosa U, Vooder T, Kolde R, Fischer K, Valk K, Tonisson N, Roosipuu R, Vilo J, Metspalu A, Annilo T (2011). Identification of miR-374a as a prognostic marker for survival in patients with early-stage nonsmall cell lung cancer. Genes Chromosomes Cancer.

[18] Han L, Wang W, Ding W, Zhang L. MiR-9 is involved in TGF-beta1-induced lung cancer cell invasion and adhesion by targeting SOX7. J Cell Mol Med. 2017;21(9):2000–08. 10.1111/jcmm.13120 PMC5571535 28266181

[19] Zeng YE, Yao XH, Yan ZP, Liu JX, Liu XH (2016). Potential signaling pathway involved in sphingosine-1-phosphate-induced epithelial-mesenchymal transition in cancer. Oncol Lett.

[20] Zeng Y, Yao X, Chen L, Yan Z, Liu J, Zhang Y, Feng T, Wu J, Liu X (2016). Sphingosine-1-phosphate induced epithelial-mesenchymal transition of hepatocellular carcinoma via an MMP-7/ syndecan-1/TGF-β autocrine loop. Oncotarget.

[21] Citron F, Armenia J, Franchin G, Polesel J, Talamini R, D'Andrea S, Sulfaro S, Croce CM, Klement W, Otasek D (2017). An integrated approach identifies mediators of local recurrence in head and neck squamous carcinoma. Clin Cancer Res.

[22] Wang H, Zhang W, Zuo Y, Ding M, Ke C, Yan R, Zhan H, Liu J, Wang J (2015). miR-9 promotes cell proliferation and inhibits apoptosis by targeting LASS2 in bladder cancer. Tumour Biol.

[23] Sondermann A, Andreghetto FM, Moulatlet AC, da Silva Victor E, de Castro MG, Nunes FD, Brandao LG, Severino P (2015). MiR-9 and miR-21 as prognostic biomarkers for recurrence in papillary thyroid cancer. Clin Exp Metastasis.

[24] Xu G, Shao G, Pan Q, Sun L, Zheng D, Li M, Li N, Shi H, Ni Y (2017). MicroRNA-9 regulates non-small cell lung cancer cell invasion and migration by targeting eukaryotic translation initiation factor 5A2. Am J Transl Res.

[25] Lee CY, Shin S, Lee J, Seo HH, Lim KH, Kim H, Choi JW, Kim SW, Lee S, Lim S, et al. MicroRNA-mediated down-regulation of apoptosis signal-regulating kinase 1 (ASK1) attenuates the apoptosis of human Mesenchymal stem cells (MSCs) transplanted into infarcted heart. Int J Mol Sci. 2016;17 10.3390/ijms17101752 PMC5085777 27775615

[26] Nie K, Gomez M, Landgraf P, Garcia JF, Liu Y, Tan LH, Chadburn A, Tuschl T, Knowles DM, Tam W (2008). MicroRNA-mediated down-regulation of PRDM1/Blimp-1 in Hodgkin/reed-Sternberg cells: a potential pathogenetic lesion in Hodgkin lymphomas. Am J Pathol.

[27] Nass D, Rosenwald S, Meiri E, Gilad S, Tabibian-Keissar H, Schlosberg A, Kuker H, Sion-Vardy N, Tobar A, Kharenko O (2009). MiR-92b and miR-9/9* are specifically expressed in brain primary tumors and can be used to differentiate primary from metastatic brain tumors. Brain Pathol.

[28] Sandoval J, Diaz-Lagares A, Salgado R, Servitje O, Climent F, Ortiz-Romero PL, Perez-Ferriols A, Garcia-Muret MP, Estrach T, Garcia M (2015). MicroRNA expression profiling and DNA methylation signature for deregulated microRNA in cutaneous T-cell lymphoma. J Invest Dermatol.

[29] Lujambio A, Calin GA, Villanueva A, Ropero S, Sanchez-Cespedes M, Blanco D, Montuenga LM, Rossi S, Nicoloso MS, Faller WJ (2008). A microRNA DNA methylation signature for human cancer metastasis. Proc Natl Acad Sci U S A.

[30] Hildebrandt MA, Gu J, Lin J, Ye Y, Tan W, Tamboli P, Wood CG, Wu X (2010). Hsa-miR-9 methylation status is associated with cancer development and metastatic recurrence in patients with clear cell renal cell carcinoma. Oncogene.

[31] Ma L, Young J, Prabhala H, Pan E, Mestdagh P, Muth D, Teruya-Feldstein J, Reinhardt F, Onder TT, Valastyan S (2010). miR-9, a MYC/MYCN-activated microRNA, regulates E-cadherin and cancer metastasis. Nat Cell Biol.

[32] Xu T, Liu X, Han L, Shen H, Liu L, Shu Y (2014). Up-regulation of miR-9 expression as a poor prognostic biomarker in patients with non-small cell lung cancer. Clin Transl Oncol.

[33] Wang R, Chen XF, Shu YQ (2015). Prediction of non-small cell lung cancer metastasis-associated microRNAs using bioinformatics. Am J Cancer Res.

[34] Polley E, Kunkel M, Evans D, Silvers T, Delosh R, Laudeman J, Ogle C, Reinhart R, Selby M, Connelly J, et al. Small cell lung cancer screen of oncology drugs, investigational agents, and gene and microRNA expression. J Natl Cancer Inst. 2016;108 10.1093/jnci/djw122 PMC6279282 27247353

[35] Chen X, Zhu L, Ma Z, Sun G, Luo X, Li M, Zhai S, Li P, Wang X (2015). Oncogenic miR-9 is a target of erlotinib in NSCLCs. Sci Rep.

